# Analytical and Numerical Investigation of the Behavior of Engineered Cementitious Composite Members under Shear Loads

**DOI:** 10.3390/ma15134640

**Published:** 2022-07-01

**Authors:** Preethy Mary Arulanandam, Madappa VR Sivasubramnaian, Maheswaran Chellapandian, Gunasekaran Murali, Nikolai Ivanovich Vatin

**Affiliations:** 1 Department of Civil Engineering, National Institute of Technology Puducherry, Karaikal 609609, Puducherry, India; preethimary1995@gmail.com; 2 Department of Civil Engineering, Mepco Schlenk Engineering College, Sivakasi 626005, Tamil Nadu, India; chellapandian@mepcoeng.ac.in; 3 Peter the Great St. Petersburg Polytechnic University, 195251 Saint Petersburg, Russia; vatin@mail.ru; 4 Division of Research & Innovation, Uttaranchal University, Dehradun 248007, Uttarakhand, India

**Keywords:** damage plasticity model, ECC, numerical modeling, shear behavior

## Abstract

This research discusses the performance of engineered cementitious composite (ECC) beams with and without transverse reinforcements using thorough analytical and finite element (FE) approaches under shear. The overall goal of this investigation was to assess the impact of various design characteristics, such as (i) shear span-to-effective depth ratio, (ii) transverse reinforcement ratio, etc., on the shear behavior of ECC beams. Nonlinear three-dimensional (3-D) FE analysis was performed with the commercial software ABAQUS to simulate the shear performance of ECC beams by employing the material properties obtained from the damage plasticity model. The correctness of the proposed FE model was validated with the benchmark experiments available in the literature. The developed FE model accurately computed the ECC beam’s overall load–deflection behavior and failure modes. In addition, the provision available in the Architectural Institute of Japan (AIJ) A-method was successfully employed to assess the shear load-carrying capacity of ECC beams. Furthermore, the effects of transverse reinforcement (*p_w_*) and shear span-to-depth ratio (a/d) on the behavior of ECC beams were also investigated. From a detailed parametric study, it was understood that a decreased a/d ratio exhibits enhanced load-carrying capacity for beams with and without stirrups for a particular cross-section. It was also observed that for the entire a/d ratio, the amount of stirrups had no substantial effect on the load-carrying capability of ECC beams.

## 1. Introduction

Engineered cementitious composite (ECC) materials are high-performance fiber-reinforced cementitious composites developed for high-tensile load-bearing applications and cost-conscious construction sectors [1,2]. To accomplish a steady-state multiple cracking and strain-hardening behavior under tension, ECC is a cement-based composite material reinforced with polymeric fibers with a dosage of 2%, such as polyethylene fibers (PE), polyvinyl alcohol (PVA), polypropylene (PP), and others. Systematic microstructure tailoring and materials optimization [3,4,5] can be used to obtain good strain-hardening behavior. The elastic modulus of ECC is lower than that of conventional concrete owing to the unavailability of coarse aggregate. However, ECC has a compressive load capacity comparable to or greater than that of regular cement concrete, with compressive strengths and strain capacities ranging from 30 to 90 MPa and 0.45–0.65 percent, respectively [6,7,8]. Similarly, ECCs possess a high tensile strength ranging from 5.0 to 8.0 MPa, corresponding to a failure strain of 3–5 percent. High strength and high modulus PE and PVA fibers were used in most structural and retrofit ECC applications mentioned in the literature [9,10]. ECC often uses a considerable volume of fine aggregate (i.e., silica sand) instead of coarse aggregate to ensure optimum stiffness and volume stability in the mix [11]. ECCs are also engineered to have a high damage tolerance and higher durability qualities when exposed to harsh environments [12].

Because of its brittle failure nature, researchers have always been interested in the shear behavior of typical concrete beams [13]. The shear span-to-depth ratio, transverse reinforcement ratio, concrete strength, section form, and structural element type are all elements that influence shear behavior. As a result, predicting shear capacity is difficult since reactions vary from section to section along the shear span [14].

Previously, Kanda and Watanabe [15] detailed the design concept and material characteristics for ECC and established its efficacy in improving structural element performance. When shear compression was prevalent, the authors found that ECC greatly improved the shear resistance of short-span beams. The ECC performed much better in the case of dominating shear stress when both deformation and shear resistance were significantly improved. The shear behavior of ECC beams with and without steel reinforcement was studied by Shimizu et al. [16]. The main parameters tested were the transverse reinforcement ratio (0%, 0.15%, and 0.30%) and the volume fraction of PVA fibers (1.0%, 1.5%, and 2.0%). According to the findings, an ECC beam with 0.30% steel reinforcement in the tension face and a fiber volume fraction of 2.0% could enhance the load-carrying capacity by a better degree than the other beams. Zhang et al. [17] analyzed the diagonal shear–tension failure characteristics in SHCC (strain-hardening cementitious composite) beams with a/d ratios of 2 and 3. A three-point bending test was performed to confirm the failure mode of beams as diagonal shear–tension mode. Moreover, by assuming that the crack surface is normal, a finite element (FE) model was created to explore shear transmission mechanisms. Singh and Sivasubramaninan [18] studied the behavior of shear critical beams made of concrete and ECC. They concluded that the ECC beam had significantly higher ductility than the typical concrete beam. Xu et al. [19] examined the shear performance of reinforced ultrahigh-toughness cementitious composite (RUHTCC) beams without transverse reinforcement. The ratio of longitudinal reinforcement and shear span–effective depth were the variables. Compared to reinforced concrete beams, RUHTCC narrow beams had higher shear strength, according to the study. In all RUHTCC beams, numerous diagonal modes and a continuous diagonal crack growth mechanism were also observed. The study also developed empirical equations for computing the ultimate shear strength of fiber-reinforced beams without stirrups, which were determined to be in line with test findings.

Zhang et al. [20] experimentally investigated the shear performance of PP-ECC beams with various shear reinforcement ratios and compared them using theoretical equations using the Japan Society of Civil Engineering (JSCE) algorithm. According to the study, the shear carrying capacity of beams with and without shear reinforcements improved by 20.6 and 107.6%, respectively, compared to that of identical concrete beams. Furthermore, the JSCE code was found to overestimate the shear capability of PP-ECC beams. Hou et al. [21] investigated RUHTCC thin-beam shear characteristics experimentally with varying web reinforcement ratios. According to the research, for RUHTCC beams with a longitudinal reinforcement ratio of 3.25 percent, minor web reinforcing may change brittle shear to ductile flexure shear behavior. The stirrup ratio was raised to 0.55 percent even for the beam with a balanced reinforcement ratio and flexural failure was obtained. The ultimate shear strength of the RUHTCC stirrup beam is somewhat higher than that of the RUHTCC beam without a stirrup. Due to stirrup limitations, the shear contribution of UHTCC decreased as the web reinforcement ratio increased. Kanakubo et al. [22] worked on the shear performance of DFRCC (ductile fiber-reinforced cementitious composite) coupling beams through anti-symmetrical bending moment loading. The parameters studied were the fiber type and fiber volume fraction. The study reported that the shear capacities of DFRCC beams with higher volume fractions could be evaluated by adding the effect of fiber carrying capacity and assumed that fiber bridging in the case of lower fiber volume fractions starts decreasing before reaching the shear capacity of the beam. Said et al. [23], with limited changes in shear span-to-depth ratio and shear reinforcement quantity, explored the shear behavior of steel-reinforced PVA-reinforced mortar-based beams. According to the study, the presence of PVA fibers is particularly significant with lower shear reinforcement, and reducing shear span-to-depth enhances beam shear capacity.

## 2. Research Significance

From a careful review of the literature, it can be learned that no previous studies have reported the effect of shear span-to-depth ratio and transverse reinforcement ratios on the behavior of ECC beams in shear. Greater variations greatly influence the performance and load-carrying capacity of beams in shear span-to-depth ratio and shear reinforcement. As a result, the current research investigation fills a knowledge gap by conducting a full numerical and analytical analysis of the shear load-carrying capability of steel-reinforced ECC beams with wider shear span-to-depth ratios and transverse reinforcement ratios. The objective of the proposed work is twofold:

- To develop a reliable and robust three-dimensional FE procedure capable of predicting the shear behavior of ECC beams using existing models.
- To determine whether the existing AIJ A-method approach for determining the load-carrying capacity of ECC beams in shear is suitable.

## 3. Experimental Corroboration

To mimic the shear performance of ECC beams with steel reinforcement, a total of seven specimens were selected from two experimental studies conducted by different researchers [20,24]. The beams were chosen with different geometry and shear span and different stirrup spacing, giving a wide range of transverse reinforcement ratios of 0–0.42%. These beams were also chosen since results such as load–deflection response and cracking patterns were readily available. The characteristics of the beam specimens are demonstrated in Table 1.

**Figure 1:**
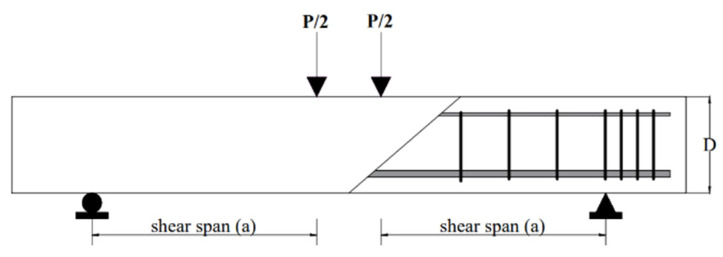
Loading pattern of the ECC beams.

Four-point loading was applied to all beam specimens, and shear failure was recorded in all beams. The loading pattern of the beams is shown in Figure 1. A linear variable displacement transducer (LVDT) was utilized to record the mid-span deflection of the beams.

## 4. Modeling of Specimens

Using the ABAQUS platform, a 3D nonlinear FE technique was created to investigate the shear behavior of ECC beams. ABAQUS is a multipurpose analysis software that can solve both linear and nonlinear problems. The selected beams’ load-versus-mid-span deflection behavior was utilized to verify the analytical results compared to the experimental data.

### 4.1. Characterization of the Material for Modeling

**Figure 2:**
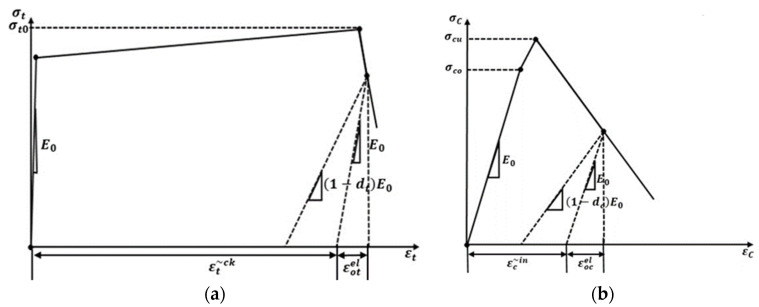
Assumed stress–strain model for ECC, (**a**) Tension, (**b**) and Compression.

The damage plasticity model in simulation software may be used to investigate the nonlinear and complicated characterization of ECC’s material constitutive behavior. The damage plasticity model is based on the model of Lubliner et al. [25], which was further developed by Lee and Fenves [26]. The damage plasticity model defines materials’ inelastic and fracture behavior by combining isotropic damage elasticity with isotropic tension and compression plasticity. It allows strain hardening, softening, uncoupled damage initiation, and buildup in tension and compression. ECC’s uniaxial tensile and compressive stress–strain behavior is used to establish the damage plasticity model. The different fiber volume fractions of ECC will influence the material behavior in tension and compression. Hence, the modeling parameters were derived from the experimental stress–strain responses (both tension and compression) for simulations incorporating the fiber volume effects. Furthermore, no fiber crack bridging was simulated as the study concentrates on the effect of shear reinforcement and shear span. The stress–strain relationship for both uniaxial tension and compression is displayed in Figure 2.

The compression and tension damage parameters ($d_{c}$ and $d_{t}$) were calculated based on Equations (1) and (2), respectively [27].

(1) $$d_{c}=1-\frac{\sigma_{c}E_{c}^{-1}}{\varepsilon_{c}^{pl}\left(1/b_{c}-1\right)+\sigma_{c}E_{c}^{-1}}$$

(2) $$d_{t}=1-\frac{\sigma_{t}E_{c}^{-1}}{\varepsilon_{t}^{pl}\left(1/b_{t}-1\right)+\sigma_{t}E_{c}^{-1}}$$

where:

$\sigma_{c}$ and $\sigma_{t}$ are compressive stress and tensile stress, respectively;

$E_{c}$—modulus of elasticity;

$\varepsilon_{c}^{pl}$ and $\varepsilon_{t}^{pl}$ are plastic strains corresponding to compressive and tensile strengths, respectively;

$b_{c}=\frac{\varepsilon_{c}^{pl}}{\varepsilon_{t}^{in}}$, $\varepsilon_{c}^{in}$—compressive inelastic strain, $b_{t}=\frac{\varepsilon_{t}^{pl}}{\varepsilon_{t}^{cr}}$, $\varepsilon_{t}^{cr}$—tensile cracking strain;

$b_{c}$ and $b_{t}$ are the constant parameters that can vary from 0 to 1, where 1 means no damage and 0 means total damage.

Experimentally acquired mechanical properties from the literature [20,24] for ECC, such as uniaxial tensile and compression stress–strain interaction performance, Young’s modulus, and Poisson’s ratio, were fed as input data to the software. The performance of steel reinforcement bars was characterized by a perfect elastic–plastic relationship, the parameters of which can also be found in other experiments [20,24]. Table 2 shows the tensile and compressive strength of the ECC employed in this investigation.

The plasticity parameters, which were taken into account for the FE analysis, are tabulated in Table 3. These plasticity parameters are values that have been found to give suitable results [27].

### 4.2. Geometrical Model

**Figure 3:**
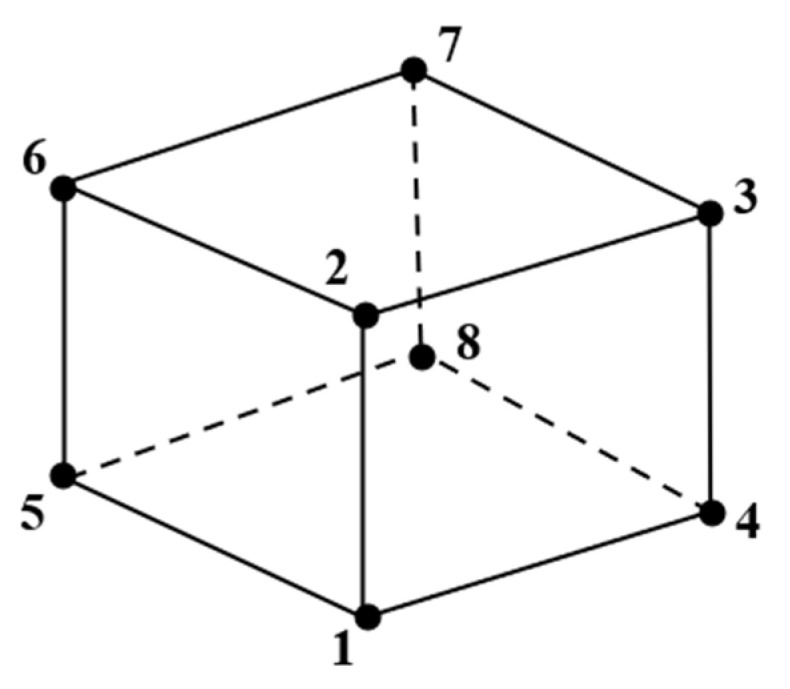
Eight-noded linear 3D brick solid element with reduced integration (C3D8R).

A linear three-dimensional (3D) brick solid element with eight nodes and reduced integration was used to model the test specimens incorporating ECC and longitudinal steel reinforcement (C3D8R), which has three degrees of freedom per nodal point (Figure 3).

**Figure 4:**
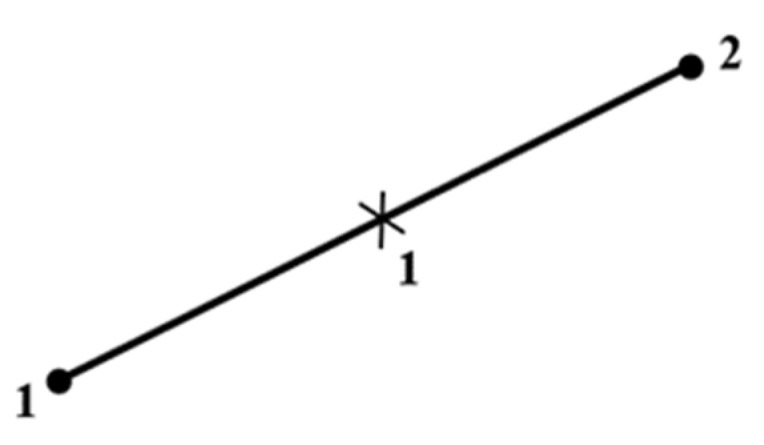
Linear 3D truss elements with two nodes (T3D2).

The brick element enables the definition of steel reinforcement using discrete rebars, so that we could model ECC with or without reinforcing rebars. Reduced integration was chosen because the integration was performed on a single integration point, which reduces the running time. The stirrup steels were modeled using two-noded linear 3D truss elements (T3D2), with three translational degrees of freedom in the nodal x, y, and z directions (Figure 4). The reinforcing elements can replicate stiffening, stress, swelling, creep, plasticity, and substantial deformations. The cross-sectional area was used to define the truss element [33,34].

**Figure 5:**
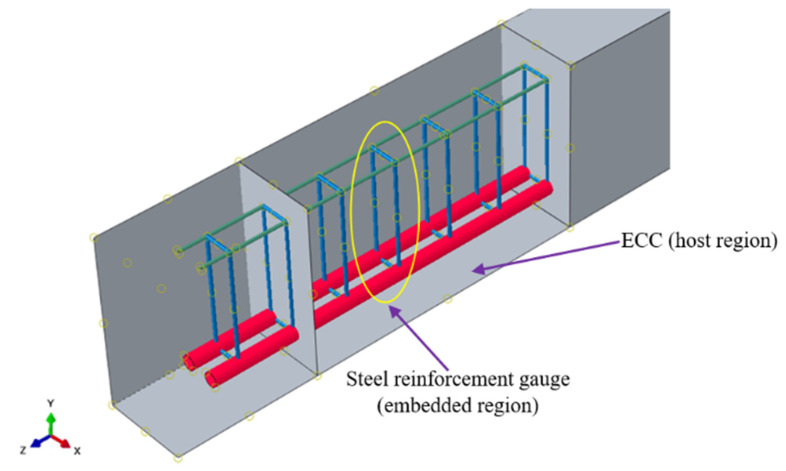
Embedded constraints used in FE Model.

The embedded constraint was used to model the perfect bond between ECC and the reinforcements in the interaction between steel reinforcement and ECC (Figure 5). Steel reinforcement bars are employed as embedded regions in this constraint, whereas ECC serves as the host region. The influence of bond–slip is ignored in the embedded region modeling method, and it is considered when determining the tensile stiffening behavior of concrete [35,36,37].

**Figure 6:**
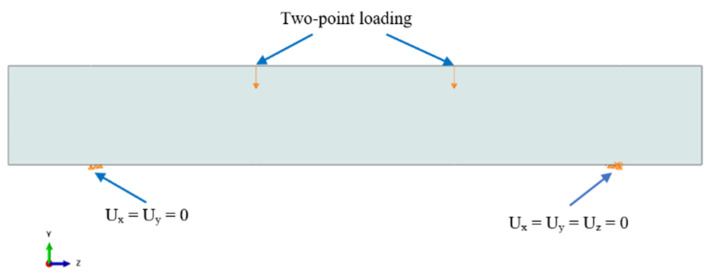
Typical loading and boundary conditions.

Modeling boundary conditions similar to experiments is essential to achieve close predictions with the experimental results. In the experiments, simply supported boundary conditions were employed with roller support at one of the ends (U_x_, U_y_) and a hinge at the other end (U_x_, U_y_, U_z_). The loads were inputted as the vertical displacement at the two loading points. The loading and boundary conditions are displayed in Figure 6.

**Figure 7:**
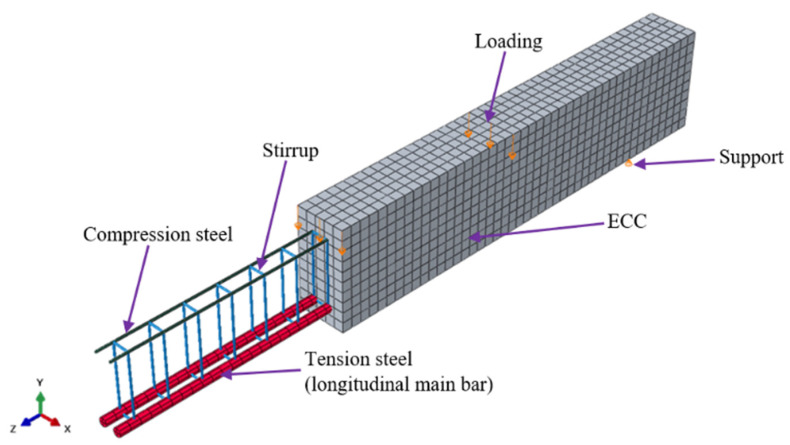
Meshing of elements used.

The FE model in this work used several mesh sizes to produce closure numerical findings in good agreement with experimental observations. This study used a relatively fine mesh size of 30 mm because it provided good accuracy with the experimental results. All elements (ECC, longitudinal, and stirrups reinforcement) were meshes with the same element size (i.e., node compatibility) to ensure that the two touching portions had common nodes. Next, the element type in ABAQUS controls the element characteristics of the mesh. For mesh control and the type of mesh, hexahedron element and structured meshing, respectively, were selected because the geometry of the element used in modeling the beam specimens was not complex [38,39]. The ECC beam that was meshed is presented in Figure 7.

**Figure 8:**
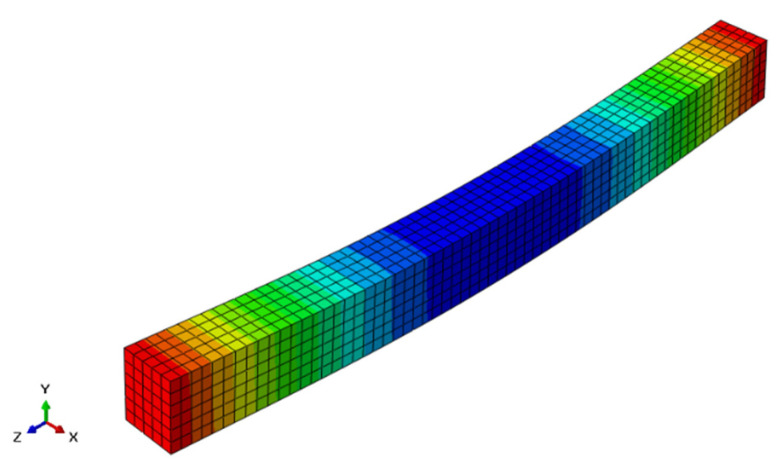
Typical deflected shape of ECC beam (units: mm).

An incremental displacement control program was adopted to analyze the ECC beams. For this, the experimental ultimate displacement value (from [20,24]) was used as the input. A nonlinear analysis divides the total specified loads on a finite element body into several load increments. The structure will be in rough equilibrium after each increment, and its stiffness matrix will be monitored for nonlinear stiffness increases. The numerical analysis is finished when the maximum displacement value is reached [40]. The typical deflected shape of the ECC beam is displayed in Figure 8.

## 5. Results and Discussion

### 5.1. Results on Relation between Load and Displacement

**Figure 9:**
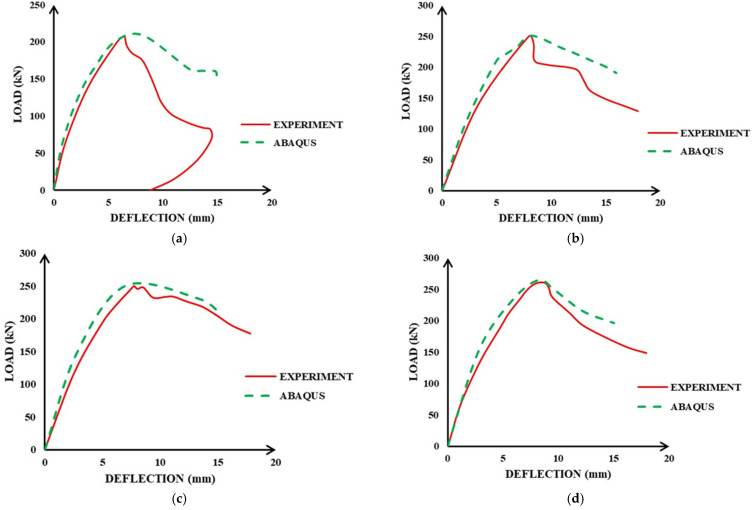

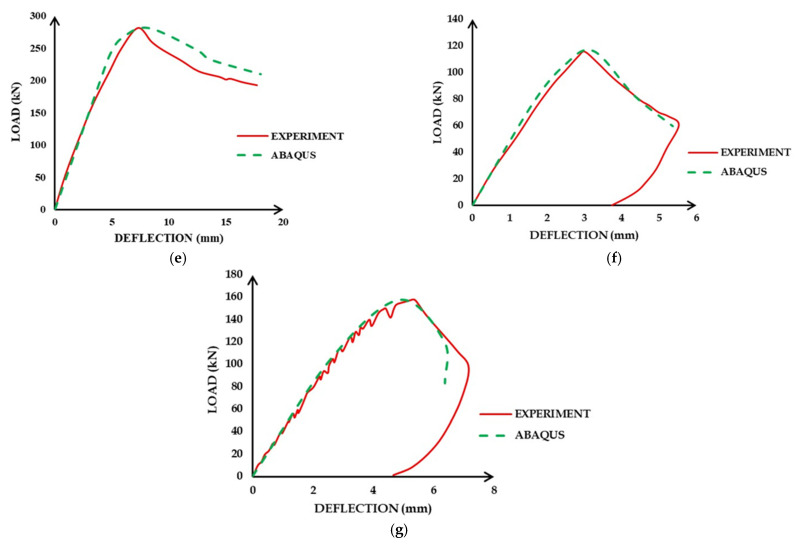
Response of ECC beams comparison under load and deflection: (**a**) P1, (**b**) P2, (**c**) P3, (**d**) P4, (**e**) P5, (**f**) P6, and (**g**) P7.

In Figure 9, we present the results for the relationship between the load–deflection obtained from the simulation for seven beam setups adopted from previous works.

The results showed variation in deflection behavior with the difference in intensity of loading, which was due to the difference in reinforcement ratios and the property of the materials used in ECC. To validate the results achieved from the FE analysis, a comparison was made with the experimental results presented in the same research works. Figure 9 depicts the comparison of modeling and experimental findings for several specimens, such as P1–P7, with changing steel reinforcement percentages and various volumetric proportions of fibers.

The ECC beams’ load–deflection characteristics were initially linear up to the cracking load, with cracks appearing in the beams’ mid-span and the curves departing from the original linear course. The load declined steadily from the peak to a constant load level for each beam, and the curve showed a softening tail after the peak. In both numerical and experimental responses, this pattern was identical to the load-deflection relationship and is displayed in Figure 9.

The stiffness responses calculated from the numerical load–deflection relationship were higher than the experimental ones. There could be many reasons behind the higher stiffness in the FE analysis. The most important reason is the development of micro-cracks due to drying shrinkage, handling, environmental effects, and so on, in the case of the experiments. Such micro-cracks are not included in FE simulations. Another cause for the simulation’s stronger initial load–deflection response could be how rebar bonds were approximated. The embedded region restriction utilized to represent the ECC–steel interaction replicates a perfect bond, as indicated previously in the geometry model. Since this bond, in reality, is not perfect, this idealization could potentially contribute to the numerical models’ initial higher stiffness. Though more research into this anomaly is needed, the mid-span displacement and ultimate load are the most critical characteristics of the current study, which were well anticipated by the FE analysis. Furthermore, the numerical load–deflection response closely matched the experimental response, demonstrating the robustness of the numerical modeling approach used.

### 5.2. Ultimate Capacity and Failure Pattern

The ultimate capacities for load and deflections and the manner of failure were determined using simulations for the tested beams in FE software. Table 4 shows that the FE model could predict the kind of failure for experimental beams with variable transverse reinforcement and a/d ratios.

The findings of the FE analysis and the experimental results demonstrated a good agreement in the ultimate capacities. Steel-reinforced ECC beams had a calculated-versus-experimental load difference of less than 3%. The average ratio of *P_u, num_/P_u, exp_* for the investigated beams was 1.01, with a standard deviation and coefficient of variation of 0.01 and 0.47%, respectively. It is clear from the comparisons that the FE procedure adopted is accurate and may be used to estimate the behavior of an ECC beam under shear.

### 5.3. Crack Pattern

**Figure 10:**
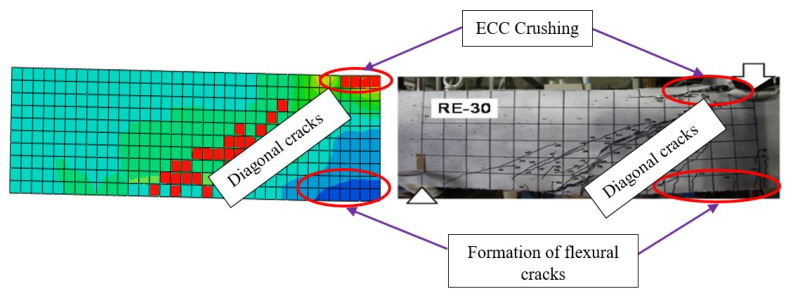
Comparison for component P4: the FE simulation’s plastic strain distribution and the experiment’s crack pattern were used.

In the damage plasticity model, cracking was assumed to begin when the maximum principal plastic strain was positive. Because the model lacks a representation for the formation of cracks at the site of material integration [25], this assumption was made. For the ECC beam P4 [20], which failed due to shear tension failure, Figure 10 compares the FE-generated plastic strain distribution and the experimentally observed fracture patterns. Figure 10 shows that the cracking patterns correlated with the experimental crack distributions up to the point of failure. Initially, the formed cracks were vertically propagating flexural cracks (blue-colored contours). As a result of increasing loading, several diagonal cracks appeared in the shear span, appearing as extensions of flexural cracks (green-colored contours).

### 5.4. Ultimate Shear Capacity of ECC Beams

**Figure 11:**
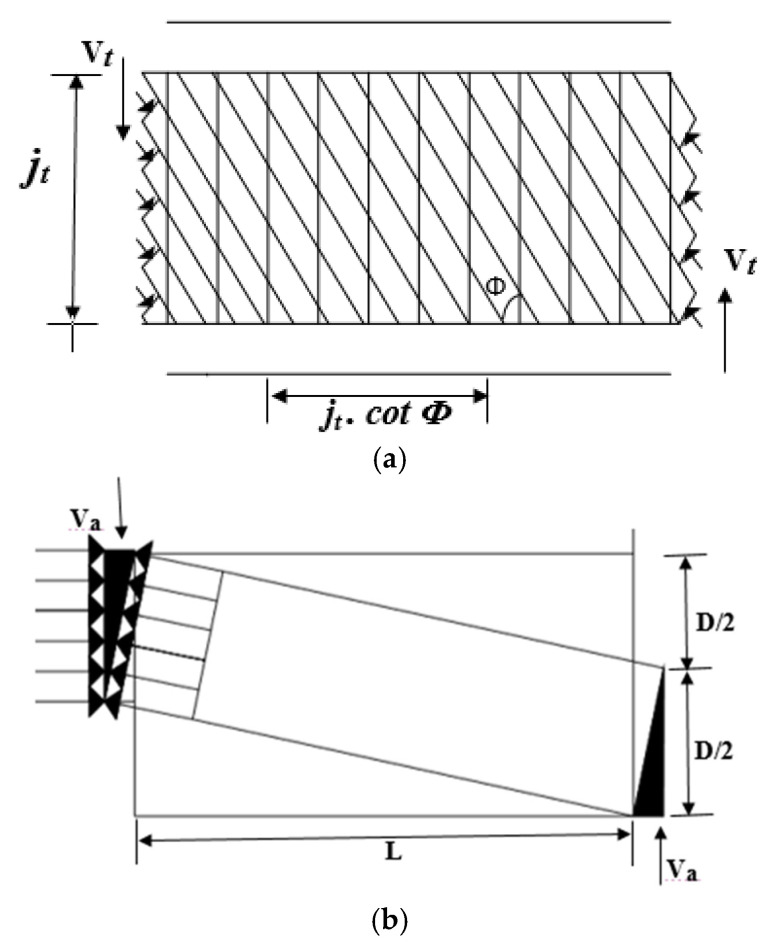
Truss and arch mechanisms based on the AIJ A-method: (**a**) truss mechanisms; (**b**) arch mechanisms.

The AIJ A-method [41,42] was accustomed to forecasting the ultimate shear load-carrying capacity of the ECC beams, which was then compared with the experimental load-carrying capacities from prior investigations. The original models were based on the plasticity hypothesis, which states that shear capacity can be depicted by superimposing truss and arch mechanisms. The employed mechanisms and the equations for predicting the ultimate shear load-carrying capacity from the adopted method follow Figure 11 and Equation (3).

(3) $$V_{su}=V_{t}+V_{a}+V_{f}$$

where $V_{su}$ = shear capacity; $V_{t}$ = shear capacity of truss mechanisms; $V_{a}$ = shear capacity of arch mechanisms; and $V_{f}$ = shear capacity by fiber bridging, in which

(4) $$V_{t}=b\cdot j_{t}\cdot p_{w}\cdot \sigma_{wy}\cdot cot\phi$$

b = member width; $j_{t}$ = distance between the top and bottom reinforcement bars; $p_{w}$ = stirrup ratio; $\sigma_{wy}$ = yield strength of stirrup. Since, the stirrups were not yielded, $\sigma_{wy}=E\cdot \varepsilon$; E= Young’s modulus of stirrup; ε= strain in the stirrup; *ϕ* = compressive strut angle that is in equilibrium with transverse reinforcement tensile force.

(5) $$V_{a}=\frac{tan\theta \cdot \left(1-\beta \right)\cdot v\cdot \sigma_{B}\cdot b\cdot D}{2}$$

(6) $$tan\theta =\sqrt{{\left(\frac{L}{D}\right)}^{2}+1}-\left(\frac{L}{D}\right)$$

(7) $$\beta =\frac{\left(1+cot^{2}\phi \right)\cdot p_{w}\cdot \sigma_{wy}}{v\cdot \sigma_{B}}\;=1.70\sigma_{B}^{-0.333}$$

$\sigma_{B}$ = compression strength of ECC; θ = arch mechanism compressive strut angle with maximum shear force; v = effective coefficient of compressive strength of ECC; D = member depth; L = test span; $v_{t}$ = reduction factor for tensile load-carrying capacity of ECC; $\sigma_{t}$ = ultimate tensile capacity of ECC; β = truss strut compressive stress-to-lowered concrete strength ratio cost, which should have the lowest value among these:

(8) $$cot\phi =min\left\{2.0,\frac{j_{t}}{Dtan\theta },\;\sqrt{\frac{v\cdot \sigma_{B}}{p_{w}\cdot \sigma_{wy}}-1}\right\}$$

To obtain the maximum value of shear force given by truss mechanisms compared to arch mechanisms, the value of *cot*ϕ should be in the region of 1.0–2.0.

(9) $$V_{f}=b\cdot j_{t}\cdot v_{t}\cdot \sigma_{t}\cdot cot\phi$$

The shear capacity increases in this technique as the capacity offered by the truss mechanisms increases with a rise in the stirrup ratio and yield strength of stirrup reinforcement used. However, arch mechanism shear capacity reduces, which is called “A-method”, whereas the “B-method” methods use a constant value of 1 for cotϕ. The ECC beams’ ultimate shear load-bearing capability was computed and shown in Table 5 using the formulae mentioned above.

Table 5 compares the experimental and predicted ultimate shear load-bearing capability of ECC beams. The results show that regardless of transverse reinforcement ratios, the AIJ A-method accurately predicted the final shear capacity of the ECC beams. The technique accurately predicted the shear capacity of other beams, except for specimens P6 and P7, which could be due to variances in the failure modes of the beam specimens. The beam specimens were subjected to flexural shear in the laboratory, while the developed model was for diagonal shear tension. It is also understood that, in most circumstances, the shear contribution of ECC fibers diminishes when shear reinforcement increases due to the sliding effect, which the AIJ A-method code ignores. The total average value of the ratio *V, _theo_*/*V, _exp_* for a beam with varied a/d ratios and shear reinforcement was 0.952, with a standard deviation of 0.33. According to the findings, the AIJ A-method code could not estimate the ultimate shear capacity of the ECC beams, regardless of the quantity of shear reinforcement used, as stated by Zhang et al. [20].

## 6. Parametric Study

Using the computational technique developed previously for verification purposes, the shear behavior of ECC structural elements with and without shear reinforcement was investigated in the range of a/d ratios from 1 to 3. Table 6 shows the a/d and transverse reinforcement ratios and the beam specimen characteristics.

**Figure 12:**
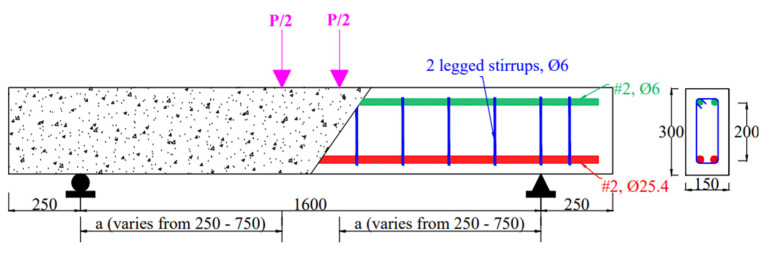
Dimensions of the ECC beam used for parametric analysis (note: all dimensions are in mm).

The adopted beam geometry and the reinforcement details were taken from the experimental investigation [20], which was validated in the earlier section. Figure 12 depicts the cross-sectional configuration of the beams employed in the parametric research and the reinforcing details.

**Figure 13:**
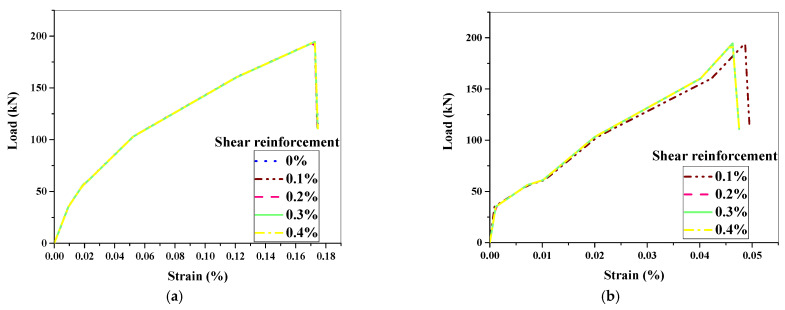
Typical load–strain response: (**a**) longitudinal main bar at mid-span; (**b**) stirrups.

To begin, Figure 13 and Table 7 show the load–strain analysis and stirrup shear resistance against shear reinforcement for all a/d ratios. The effects of the a/d ratio on specific shear reinforcement and the effects of shear reinforcement on a specific a/d ratio were investigated.

### 6.1. Analysis of Load–Strain Relationship

The load–strain response of longitudinal bars was seen and monitored at mid-span using the simulation of beams. The typical load–strain response of the longitudinal main bars is shown in Figure 13a. Furthermore, the highest strain at ultimate load was 0.18 percent, indicating that the longitudinal main bars yielded, as Zhang et al. [20] stated. Furthermore, the strains at maximal load in all stirrups were measured, and they were determined to be 0.10 percent, indicating that the stirrups did not yield due to flexure. Figure 13b depicts a stirrup’s typical load–strain response. From the above-mentioned observations, it is worth noting that the beam specimens underwent flexure-dominated shear and attained failure, unlike pure shear failure, as demonstrated by Sunaga et al. [43], comprising diagonal tension and stirrup yielding.

### 6.2. Shear Span-to-Depth Ratio against Particular Shear Reinforcement

**Figure 14:**
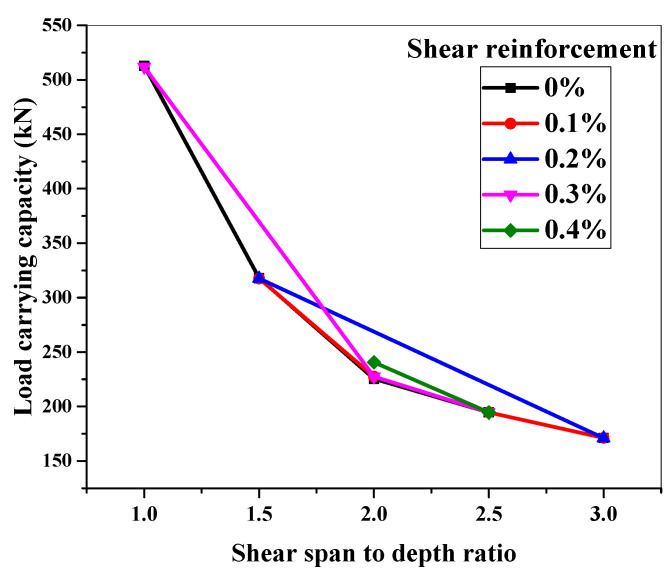
Effect of a/d ratio on the shear capacity of ECC beams.

This section investigates the shear behavior of ECC beams with and without shear reinforcement for various a/d ratios for a certain cross-section (Figure 12). For this, shear reinforcements of 0%, 0.1%, 0.2%, 0.3%, and 0.4% were chosen and tested against various a/d ratios ranging from 1 to 3, within a 0.5 percent interval (i.e., 1, 1.5, 2, 2.5, and 3). Shear reinforcements were chosen depending on the shear spans employed due to the variation in stirrup spacing. The load-carrying capability of ECC beams with and without shear reinforcement is shown in Figure 14.

Other parameters of the beams, such as depth, width, span, and longitudinal reinforcement ratio, were held constant while the a/d ratios were varied.

Figure 14 illustrates that regardless of the amount of shear reinforcement for a given cross-section, increasing the a/d ratio affects the shear load-carrying capacity of ECC beams. An increase in the a/d ratio causes this influence, which shifts the failure mode from shear to flexure. Beams with an a/d ratio of 1 had twice the load-carrying capacity of beams with a/d ratios of 3 and 2.5.

### 6.3. Effect of Shear Reinforcement against Particular Shear Span-to-Depth Ratio

**Figure 15:**
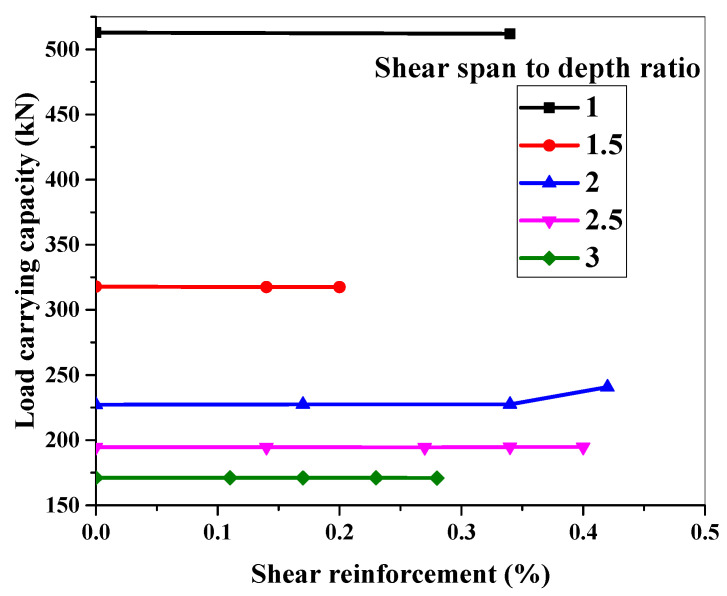
Effect of different reinforcement ratios on the shear capacity of ECC beams.

The shear behavior of the ECC beam is explored in this section for various a/d ratios against a specific shear reinforcement ratio. Figure 12 shows the adopted cross-section, a/d ratios, and shear reinforcement details for this experiment. Details of the ECC beam’s geometry are shown in Figure 12. The load-carrying capacity of the simulated ECC beams with an a/d ratio of 1 to 3 is shown in Figure 15.

The depth, width, span, and longitudinal reinforcement ratio of the beam were all held constant while the shear reinforcement was changed. As illustrated in Figure 15, the load-carrying capacity of ECC beams did not change appreciably when shear reinforcement was changed for all a/d ratios. As Zhang et al. [20] asserted, the amount of shear reinforcement for a given cross-section for all a/d ratios does not influence the load-carrying capacity of ECC beams. As a result, reducing shear reinforcement in ECC beams across the board looks to be gaining traction.

### 6.4. Crack Pattern and Failure Mode

**Figure 16:**
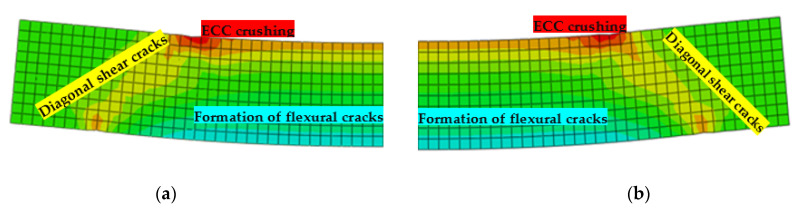
Plastic strain distribution for beams: (**a**) S-1; (**b**) S-2.

In this section, the crack pattern and the mode of failure for the beams used in the parametric study are discussed. It was noted that all beams failed with flexure-dominated shear failure. Figure 16a,b compare the plastic strain obtained from the FE analysis for S-1 (without stirrups) and S-2 (with stirrups) beam specimens, respectively, used in the parametric study. It was observed that at earlier stages, the cracks propagated in the vertical direction and as increases in load occurred, the inclined shear cracks started to appear. Finally, the failure occurred due to the crushing of ECC in diagonal cracks and in the compression zone. In addition, the number of stirrups did not have any significant effect on the crack pattern and the mode of failure. Similar behavior was achieved for all the beams.

### 6.5. Shear Capacity of Beams

This part also looks into the ECC matrix and stirrups’ shear load-bearing capabilities. Zhang et al. [20] estimated the shear load capacity of the shear reinforcement using Equation (10).

(10) $$V_{s}=\overset{n_{s}}{\underset{i=1}{\sum }}A_{w}E_{s}\varepsilon_{si}$$

where $A_{w}—$area of the stirrup; $E_{s\;}—$young’s modulus of the stirrup; $\varepsilon_{si}—$strain of the stirrup; and $n_{s}—$number of shear reinforcements crossing the critical crack. The shear load capability of the ECC matrix and stirrups is shown in Table 7. By deducting the shear reinforcement capacity from the overall shear capacity of the beam, the ECC matrix’s shear load-carrying capacity was derived. Table 7 shows that the ECC matrix provided more than 70% of the shear resistance, with stirrups accounting for the rest. As the a/d ratio grew, the ECC matrix provided improved shear resistance.

## 7. Conclusions

This study looked at the effects of the shear span-to-depth ratio and transverse reinforcement ratio on flexure-dominated shear steel-reinforced ECC beams. Using the damage plasticity model and the AI—A approach, thorough numerical modeling and an analytical analysis were carried out. The following conclusions were taken from the analysis’ findings:

- Damage parameters and a damage plasticity model from the nonlinear finite element platform can predict the overall behavior of ECC under shear-dominant loads.
- The numerical study validates the results of other researchers’ experimental investigations into load, deflection, and failure modes, which were very similar. The obtained numerical and experimental load–deflection responses exhibited close agreement with each other. Furthermore, the difference in the peak load of the numerical modeling and experimental responses of all the beams was within the range of 3%, irrespective of the amount of reinforcement and a/d ratios, which shows the robustness of the procedure adopted in the FE analysis.
- The existing AIJ A-method fairly estimated the shear capacity of ECC beams as the beams demonstrated flexure-dominated shear failure, i.e., cracking in flexure with high longitudinal stress.
- Because of the dominant shear failure, simulated reinforced ECC beams with lower a/d ratios had higher load-carrying capacities, regardless of the degree of shear reinforcement.
- Stirrups did not affect the load-carrying capabilities of ECC beams for varied a/d ratios, regardless of the transverse reinforcement ratio.

## Figures and Tables

**Table 1:** Specifications of beams.

Sl. No.	Specimen ID	Dimension (B × D × L) * (mm)	Shear Span (a) (mm)	Shear Span-to-Depth Ratio	Longitudinal Reinforcement Ratio ^#^ (%)	Transverse Reinforcement Ratio (*p_w_*) ^$^ (%)	Stirrup Spacing (mm)
1.	P1 [20]	150 × 300 ×2100	700	2.8	2.7	0	0
2.	P2 [20]	150 × 300 ×2100	700	2.8	2.7	0.12	350
3.	P3 [20]	150 × 300 ×2100	700	2.8	2.7	0.24	175
4.	P4 [20]	150 × 300 ×2100	700	2.8	2.7	0.30	140
5.	P5 [20]	150 × 300 ×2100	700	2.8	2.7	0.42	100
6.	P6 [24]	100 × 200 ×1100	267	1.53	1.14	0	0
7.	P7 [24]	100 × 200 ×1100	267	1.53	1.14	0.42	133.5

* (B = breadth, D = overall depth, and L = span); ^#^ (longitudinal reinforcement ratio (%) = (area of steel reinforcement/(breadth × height)) × 100); ^$^ (shear reinforcement ratio (%) = (area of steel reinforcement/(breadth * spacing of stirrups)) × 100).

**Table 2:** Input parameters for ECC.

Specimen ID	Tensile Strength (MPa)	Tensile Strain (%)	Compressive Strength (MPa)	Compressive Strain (%)	Young’s Modulus (GPa)	Poisson’s Ratio
P1–P5 [20]	4.0	1.2	32.7	0.8	18	0.19
P6–P7 [24]	5.1	1.7	73.0	1.1	20.4	0.20

**Table 3:** Details of plasticity parameters.

Notation	Value
Angle of dilation (*Ψ*) [28]	20
Eccentricity ratio (*ε*) [29]	0.1
Ratio of biaxial-to-axial compressive stress $(\sigma_{b0}/\sigma_{c0}$) [30]	1.16
$K_{C}$ [31]	0.67
Viscosity Coefficient (*µ*) [32]	0.01

**Table 4:** Summary of comparative study details.

Sl. No.	Specimen ID	Experimental Results			FE Results			*P_u, num_*/*P_u, exp_*	% Difference in Ultimate Load
		*P_u, exp_* (kN)	*δ_u, exp_* (mm)	Mode of Failure	*P_u, num_* (kN)	*δ_u, num_* (mm)	Mode of Failure		
1.	P1 [20]	207.8	8.19	ST	208.6	6.4	ST	1.00	0.38
2.	P2 [20]	250.2	10.02	ST	251.3	8.22	ST	1.00	0.44
3.	P3 [20]	250.1	9.87	ST	252.4	6.93	ST	1.01	0.92
4.	P4 [20]	260.4	10.55	ST	264.8	8.19	ST	1.02	1.69
5.	P5 [20]	281.2	9.23	ST	284.1	8.18	ST	1.01	1.03
6.	P6 [24]	115.7	3.00	ST	116.2	3.2	ST	1.00	0.43
7.	P7 [24]	157.3	5.2	ST	158.2	4.9	ST	1.01	0.57

*P_u_*—ultimate load; *δ_u_*—ultimate deflection; ST—shear tension.

**Table 5:** Summary of predicted ultimate load-carrying capacity.

Sl. No.	Specimen ID	Experimental Load (*V, _exp_*) (kN)	*V_su_* (*V, _theo_*) (kN)	Shear Strength Shared by			*V, _theo_/V, _exp_*
				Truss Mechanism (*V_t_*) (%)	Arch Mechanism (*V_a_*) (%)	Fiber Bridging Mechanism (*V_f_*) (%)	
1.	P1 [20]	207.8	171.8	0	47	53	1.21
2.	P2 [20]	250.2	188.5	12	40	48	1.32
3.	P3 [20]	250.1	214.7	24	32	44	1.31
4.	P4 [20]	260.4	231.9	28	29	43	1.27
5.	P5 [20]	281.2	216.9	38	21	42	1.30
6.	P6 [24]	115.7	170.1	0	58	42	0.68
7.	P7 [24]	157.3	202.8	32	33	35	0.78

**Table 6:** Details of the simulated beam specimens.

Beam	Shear Span, a (mm)	Shear Span-to-Depth Ratio (a/d)	Transverse Reinforcement Ratio (%)
S-1	250	1	0
S-2	250	1	0.3
S-3	375	1.5	0
S-4	375	1.5	0.1
S-5	375	1.5	0.2
S-6	250	2	0
S-7	250	2	0.2
S-8	250	2	0.3
S-9	250	2	0.4
S-10	625	2.5	0
S-11	625	2.5	0.1
S-12	625	2.5	0.2
S-13	625	2.5	0.3
S-14	625	2.5	0.4
S-15	750	3	0
S-16	750	3	0.1
S-17	750	3	0.2
S-18	750	3	0.3

**Table 7:** Contribution of shear load from ECC and stirrups.

Beam	Total Shear Capacity (kN)	Shear Load Carried by	
		ECC Matrix (%)	Stirrups (%)
S-1	256.5	100	-
S-2	255.9	89.4	10.6
S-3	158.9	100	-
S-4	158.7	94.3	5.7
S-5	158.7	91.5	8.5
S-6	113.7	100	-
S-7	113.7	88.1	11.9
S-8	113.7	76.1	23.9
S-9	113.7	70.2	29.8
S-10	97.2	100	-
S-11	97.2	90.7	9.3
S-12	97.2	81.4	18.6
S-13	97.3	76.8	23.2
S-14	97.3	72.1	27.9
S-15	85.5	100	-
S-16	85.4	94.7	5.3
S-17	85.5	89.4	10.6
S-18	85.4	86.8	13.2

## Data Availability

Not applicable.
